# Unravelling Prostate Cancer Heterogeneity Using Spatial Approaches to Lipidomics and Transcriptomics

**DOI:** 10.3390/cancers14071702

**Published:** 2022-03-27

**Authors:** Shadrack M. Mutuku, Xander Spotbeen, Paul J. Trim, Marten F. Snel, Lisa M. Butler, Johannes V. Swinnen

**Affiliations:** 1 Adelaide Medical School, University of Adelaide, Adelaide, SA 5005, Australia; shadymutu@gmail.com (S.M.M.); paul.trim@sahmri.com (P.J.T.); marten.snel@sahmri.com (M.F.S.); lisa.butler@adelaide.edu.au (L.M.B.); 2 South Australian Health and Medical Research Institute (SAHMRI), Adelaide, SA 5000, Australia; 3 Laboratory of Lipid Metabolism and Cancer, Leuven Cancer Institute, Department of Oncology, KU Leuven, 3000 Leuven, Belgium; xander.spotbeen@kuleuven.be; 4 South Australian Immunogenomics Cancer Institute, University of Adelaide, Adelaide, SA 5005, Australia; 5 Freemasons Centre for Male Health and Wellbeing, University of Adelaide, Adelaide, SA 5005, Australia

**Keywords:** prostate cancer, lipids, biomarkers, mass spectrometry imaging, lipidomics, metabolomics, MALDI

## Abstract

**Simple Summary:**

Prostate cancer is a heterogenous disease in terms of disease aggressiveness and therapy response, leading to dilemmas in treatment decisions. This heterogeneity reflects the multifocal nature of prostate cancer and its diversity in cellular and molecular composition, necessitating spatial molecular approaches. Here in view of the emerging importance of rewired lipid metabolism as a source of biomarkers and therapeutic targets for prostate cancer, we highlight recent advancements in technologies that enable the spatial mapping of lipids and related metabolic pathways associated with prostate cancer development and progression. We also evaluate their potential for future implementation in treatment decision-making in the clinical management of prostate cancer.

**Abstract:**

Due to advances in the detection and management of prostate cancer over the past 20 years, most cases of localised disease are now potentially curable by surgery or radiotherapy, or amenable to active surveillance without treatment. However, this has given rise to a new dilemma for disease management; the inability to distinguish indolent from lethal, aggressive forms of prostate cancer, leading to substantial overtreatment of some patients and delayed intervention for others. Driving this uncertainty is the critical deficit of novel targets for systemic therapy and of validated biomarkers that can inform treatment decision-making and to select and monitor therapy. In part, this lack of progress reflects the inherent challenge of undertaking target and biomarker discovery in clinical prostate tumours, which are cellularly heterogeneous and multifocal, necessitating the use of spatial analytical approaches. In this review, the principles of mass spectrometry-based lipid imaging and complementary gene-based spatial omics technologies, their application to prostate cancer and recent advancements in these technologies are considered. We put in perspective studies that describe spatially-resolved lipid maps and metabolic genes that are associated with prostate tumours compared to benign tissue and increased risk of disease progression, with the aim of evaluating the future implementation of spatial lipidomics and complementary transcriptomics for prognostication, target identification and treatment decision-making for prostate cancer.

## 1. Prostate Cancer Heterogeneity

Prostate cancer (PCa) is a leading cause of cancer mortality and is the second most common cancer among men in developed countries [1,2,3]. PCa is a phenotypically and molecularly heterogenous disease, which presents challenges for disease diagnosis and treatment decision-making. Phenotypically, long term follow-up studies have revealed that the majority of men diagnosed with PCa have an indolent form of disease that does not require immediate treatment and is amenable to active surveillance strategies, meaning close monitoring for cancer progression [4]. For patients uncomfortable with active surveillance, or who display clinical criteria of higher risk disease, potentially curative treatment options include radical prostatectomy (the surgical removal of the prostate gland) or radiation therapy, albeit these modalities come with common side effects that seriously affect patients’ quality of life, including incontinence and loss of erectile function [5]. The increasing implementation of prostate-specific antigen (PSA) testing in the Western world over the past three decades has led to many more cases being detected but this also contributed significantly to overtreatment of men with PCa [6]. PSA is also upregulated in non-cancerous disease states, such as prostatitis, enlarged prostate (benign prostatic hyperplasia) and infection. Thus, at the time of diagnosis, the tools are still lacking to differentiate between patients with indolent disease, those who would benefit from standard therapy or patients that might benefit from a more aggressive than standard intervention [4].

Histopathological diagnosis (classification) of prostate cancer is currently performed using the Gleason scoring (GS) system, involving assessment of architectural, nuclear, and luminal features of the prostate tumour tissue. The GS system uses a range from grade 1 (well-differentiated epithelial cells with small-sized nuclei) to grade 5 (poorly differentiated, enlarged hyperchromatic nuclei). A new grading system by the International Society of Urological Pathology uses grade groups for disease stratification, where grade group 1 is low risk and grade group 5 is the highest risk disease [7]. This key pathological tool can be prone to pitfalls, such as incorrect identification of adenocarcinoma from other mimickers, such as prostatic intraepithelial neoplasia (PIN) [8]. Together with PSA testing and pathological Gleason grading, the tumour, node, metastasis (TNM) staging system is employed for clinical decision-making in prostate cancer [9].

The heterogeneity of individual PCa clinical behaviour (i.e., indolent versus aggressive disease) likely reflects its molecular heterogeneity at a tissue and cellular level. The majority of PCas are known to be multifocal, meaning that numerous distinct tumour clones can co-exist within the same primary tumour [4,10,11]. These subpopulations of tumour cells contain different mutations, copy number variations (CNVs) and gene expression profiles, leading to the characteristic intra-tumoural heterogeneity of PCa. Spatial heterogeneity also appears to be a key feature of the tumour microenvironment (TME), with factors such as cell type, cell shape, cell–cell, and cell–extracellular matrix (ECM) interactions all being of importance to understand tissue functionality and corresponding pathological changes [12,13], and is an important determinant in cancer prognosis and susceptibility of a patient to a certain treatment [14]. Such heterogeneity in the TME requires a spatial approach to biomarker discovery and biological analysis.

## 2. Rewired Lipid Metabolism as a Source of Biomarkers and Therapeutic Targets

To minimise the overdiagnosis and overtreatment associated with the current PSA-based diagnostic tests, there is an acknowledged need for prognostic clinical and molecular biomarkers to be incorporated in the diagnosis of PCa [4,15]. Omics (e.g., genomics, transcriptomics, lipidomics, proteomics, epigenomics)-based research has played a very important role in discovering novel targets and putative biomarkers for prostate and other cancers. While genomic and transcriptomic analyses have greatly improved the stratification of patients in other cancer types, despite providing a wealth of molecular insight in PCa development and progression, these approaches have not led to the same transformation of clinical diagnostics and disease management in the case of PCa. In contrast, the ubiquitous cancer-related rewiring of metabolism that in part is driven by genetic and epigenic changes, holds great promise for the development of metabolite-based biomarkers as well as the discovery of possible druggable targets [16]. Lipids in particular appear to hold significant potential and have been studied intensively. The strong interest in lipid profiling of PCa stems from the fact that male sex steroid hormones, or androgens, which promote the development and progression of PCa, are potent regulators of lipid metabolism [17,18]. PCa cells often exhibit deregulated androgen signalling that enhances lipid metabolism through the overexpression of key lipid synthetic enzymes [19,20] and mobilisation of fatty acid uptake from the circulation or from adipose tissue in the TME by lipolysis [21,22]. Multiple studies have deduced that enhanced fatty acid synthesis and their utilisation from extracellular sources is a feature of PCa cells that promotes tumour cell proliferation, bone metastasis, and disease progression [23]. This metabolic reprogramming, together with the important roles of lipids in a wide array of physiological functions in health and disease [24], and advances in mass spectrometry based lipidomics technologies [25,26], has led to increased interest in lipid biomarker research.

## 3. The Need for Spatial Omics Approaches

Thus far, most omics analyses of PCa tissue, including lipidomics, have been applied to tissue extracts. Electrospray ionisation technology [27] has played an important role in the development of conventional lipidomics tools such as shotgun lipidomics [28,29] and liquid chromatography mass spectrometry (LC–MS) lipidomics for the analysis of lipids in cell line models [30], patient-derived tissues [31] and plasma [32,33]. Such bulk omics research has played an important role in identifying novel targets and putative biomarkers for PCa. However, biomarker detection and functional characterisation in the setting of multifocal primary prostate tumours remains a significant challenge, as bulk omics analyses represent an integrated average molecular composition of a tissue, comprising various cell types. Recent advances in single cell omics approaches have in part alleviated these issues, but overall lack the spatial information that is critical to capture localised or histology-restricted changes in cell type composition. It is only relatively recently that spatial omics approaches have been applied in the context of PCa tissues, but these are critical in view of the heterogenous nature of the prostate gland [34] and its tumours [35,36], which arise from multifocal lesions [37]. Moreover, the TME is highly heterogeneous with a dynamic interplay of several cell types, including fibroblasts, muscular, epithelial, endothelial and immune cells, each with its own characteristic lipid profile due to cell-specific enzyme expression and adaptation to a changing local TME. Hence, lipid profiles in PCa development and progression are dynamic and exhibit a high degree of cell specificity that are optimally mapped and understood using spatial approaches. Here, we will summarise the current status of this field, focusing mainly on spatial lipidomics by mass spectrometry imaging and complementary spatial transcriptomics, as these relate to the discovery of prognostic and diagnostic markers of prostate cancer.

## 4. Experimental Approaches in Spatial Lipidomics

The “lipidome” is the comprehensive composite of all lipid classes found in a cell and/or its subcellular compartments and biological fluids (plasma, serum, saliva). Lipidomics is a subset of the field of metabolomics and shares similar analytical workflows often centred on mass spectrometry. Mass spectrometry imaging (MSI) can provide spatial detail of cellular and compartmental source metabolites in tissues. Now MSI has become an increasingly popular tool for lipidomics, due to its power to detect perturbations in lipid content within heterogenous and complex biological samples [38].

**Figure 1:**
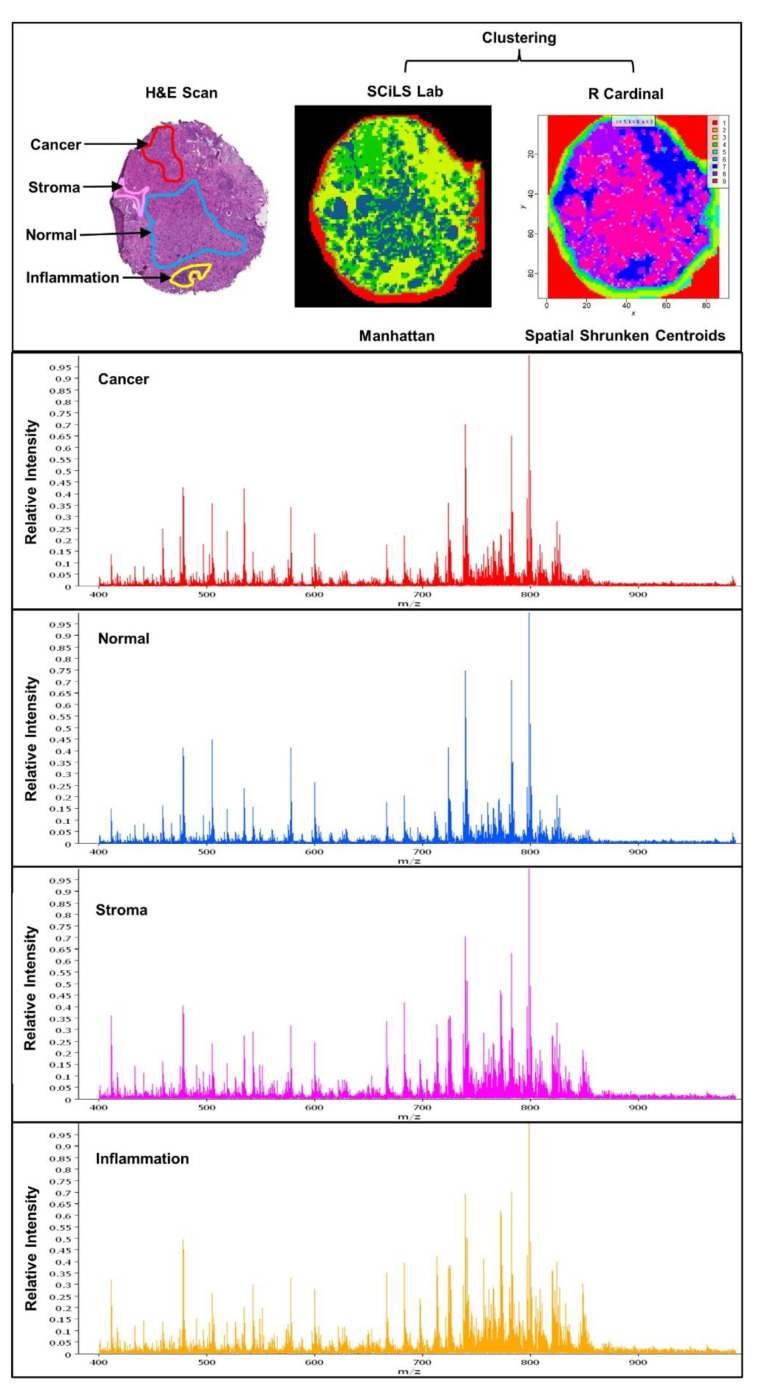
MALDI-MSI of prostate tumours. Tissue morphology (H&E digital scan) of multifocal disease in prostate tissue and spatial segmentation using clustering in SCiLS Lab and R *Cardinal* from a serial imaged section. The associated mass spectra show different *m*/*z* features for cancer—red, normal—blue spectra, stroma—pink, and inflammation—yellow. Average spectra are normalised to total ion count method.

MSI is commonly used as an untargeted tool for discovery lipidomics, with MSI sources coupled to time-of-flight (TOF), quadrupole TOF, Orbitrap, and Fourier transform ion cyclotron resonance (FT-ICR) mass analysers [39]. MSI is based on the generation of ions at definite co-ordinate loci across a tissue or inorganic surface [40]. Molecular information from multiple mass spectra is then combined to create an ion map akin to chemical histology [41]. There are multiple MSI technologies currently in use. The most widely used is matrix-assisted laser desorption ionisation (MALDI) which uses a chemical matrix that is uniformly applied to the tissue and absorbs the energy from the UV laser. The matrix ionises biomolecules and conveys them into the gas-phase [42,43]. Initial MALDI-MSI studies in the late 1990s were focused on mapping the distribution of intact proteins. This was expanded to the imaging of lipids in the 2000s [44,45,46]. Typical spatial resolutions that are achieved using this technology range between 10 µm and 100 µm, thus slightly above single cell resolution. With increasing maturation of the technology, MSI is increasingly being applied to obtaining in situ information on metabolites and lipids in many solid malignancies [38,47,48] including PCa. MALDI-MSI offers a powerful label-free semiquantitative technique to detect lipids and other biomolecules in tissue (Figure 1). Co-registration or overlay of MALDI ion maps with histological scan of tissues can link biomolecule presence and/or abundance to key anatomical and morphological features. Hence, MSI of lipids has the potential to provide information about disease aggressiveness [49], monitor disease progression and pharmacodynamic effects of therapeutic agents. In this latter context, using both MALDI MS/MS imaging and liquid chromatography tandem mass spectrometry (LC–MS/MS), the uptake kinetics of current PCa clinical agent enzalutamide were detected in patient-derived prostate explants, with the drug ion signal spatially localised to heterogenous epithelial regions, which were rich in the drug target [50].

Although the majority of the MSI work discussed in this review was produced using the MALDI method, it is worth noting that other ionisation techniques are also applicable. Another now-commonly used MSI method is called desorption electrospray ionisation (DESI) [51,52]. It employs an electrically charged solvent spray that is scanned across the tissue surface. Analyte molecules are desorbed from the surface, ionised, and entrained in the spray to be drawn into the mass spectrometer via a transfer capillary [52]. The absence of a matrix makes DESI imaging data less complex than MALDI imaging data which can make lipid assignments easier in the absence of matrix ions. There is also the advantage of doping derivatives, such as Li^+^ for tandem MS or Ag^+^ for improved detection of unsaturated olefins and cholesterol esters [53], but the lateral resolution with DESI is typically 20–200 µm which compares unfavourably to high-definition MALDI imaging. Secondary ion mass spectrometry (SIMS) is a long-established ionisation method for the analysis of solid surfaces by sputtering of the sample with a focused primary ion beam and collecting the ejected secondary ions [54,55]. SIMS is a matrix-free approach and can achieve very high spatial resolution of 100 nm, which generates spectra from sub-cellular lipidomes. It should be noted that SIMS is a much harder ionisation technique than DESI and MALDI and analyte molecules are often fragmented in source, for instance phosphatidylcholine (PC) is measured indirectly via the choline headgroup fragment, however more recent advances in SIMS have shown imaging of intact lipids [56].

Ultimately, the choice of analytical method to unravel lipidomic profiles is guided by type of sample, availability of instrument, expertise of the user, and the target molecule (s) required to answer the biological question. Spatially resolved lipidomics is now a useful addition to the PCa researcher’s arsenal. Some limitations remain, chief of which are that the technique is usually only semi-quantitative, and that confident identification of lipids is challenging. Identification is hampered by sample preparation options and limited choices of orthogonal dimensions of separation. Sample preparation methods that retain spatial integrity offer very limited ability to reduce sample complexity. Additional dimensions of separation can only by efficient if they can be applied post-ionisation. One such method is ion mobility separation, which is discussed in more detail later. A useful way to compensate for the shortfalls of spatial lipidomics is by combining it with other bioanalytical measurements performed on the same samples, e.g., transcriptomics, conventional lipidomics, proteomics, spatial transcriptomics, etc. There is however no simple way of combining complex data from multiple sources.

## 5. Spatial Lipidomics Applied to Prostate Cancer

In the field of PCa, MSI has, to date, primarily been applied toward identifying lipidomic and metabolomic phenotypes related to the presence of malignancy and the severity of tumour grade, albeit in mostly very small patient cohorts (summarised in Table 1). Initially, increased abundance of multiple phospholipid (PL) classes in PCa was reported using MALDI-MSI in a discovery patient set of tissues (*n* = 14) [57]. In this study, 14 phosphatidylinositol (PI), 3 phosphatidylethanolamine (PE), and 3 phosphatidic acid (PA) species were highly abundant in cancer, specifically PI(18:0/18:1), PI (18:0/20:3), and PI(18:0/20:2) were significantly abundant lipids (*p* value ≤ 0.05). A validation set (*n* = 24) was built using an orthogonal partial least squares discriminant analysis (OPLS-DA) model that established PI species to have 87.5% sensitivity and 91.7% specificity for PCa diagnosis [57]. The authors postulated that PI distribution may be related to changes in acyltransferase activity and PI3K signalling [57]. Following this report, Goto and colleagues showed using positive ion mode MALDI-IT-TOF imaging, increased levels of lyso-PC (LPC) (16:0) in benign epithelium compared to cancer, which was prognostic for biochemical recurrence (increasing serum PSA levels) after radical prostatectomy [58]. The enhanced LPC levels in normal tissue may reflect increased activity of lysophospholipase D activity and PC remodelling pathways. Wang and colleagues conducted metabolomic imaging of prostate tissue (*n* = 3 patients) by MALDI FT-ICR [59], using LC–MS/MS as a structural validation tool. In the study, *m*/*z* 534.296 PC (16:0) and *m*/*z* 740.520 PE (34:1) were abundant in cancerous regions whilst neutral lipids, *m*/*z* 633.485 diglyceride (DG) (34:1), *m*/*z* 895.716, triglyceride (TG) (52:3) and *m*/*z* 951.778 TG (56:3) and *m*/*z* 769.562 sphingomyelin (SM) (d36:1) were distributed in non-cancerous regions, although this might have encompassed stromal adiposity. The 9-aminoacridine (9-AA) matrix afforded the detection of nucleotide anions in which they argued *m*/*z* 505.989 ATP was enhanced in cancerous regions while *m*/*z* 346.056 AMP and *m*/*z* 426.022 ADP were diminished consistent with increased ATP flux that is critical for tumour cell proliferation [59].

In 2019, Randall and colleagues reported a study of 10 PCa specimens with varying pathological Gleason scores (GS) for analysis by MALDI FT-ICR MSI with α-cyano-4-hydroxycinnamic acid matrix in positive ion mode [60]. Three additional specimens were used for MALDI TOF MSI and 4 additional samples for liquid extraction surface analysis (LESA). They identified 481 *m*/*z* features that discriminated between GS (3 + 4) and GS (4 + 3) tumours with sensitivity and specificity analysis ROC values above a threshold of 0.75. Of fifty-six ions searched against the online Lipid Maps database (www.lipidmaps.org), tentative identifications of four PC, four PA, eight phosphatidylserine (PS), four cardiolipins (CL), and five PIs were made. However, none of the ions were classifiers of either of the two grades, albeit CL were detected more frequently in higher GS disease, consistent with a previous report [30]. Five additional specimens with tumour grades consistent with the first data set were used in a validation set by MALDI FT-ICR MSI, which resulted in similar variation in distribution of lipid *m*/*z* features. Despite the small sample size employed in this study, the investigators demonstrated the ability of MALDI-MSI to identify tumour-specific lipid markers, palmitoylcarnitine and stearoylcarnitine, which were detected as discriminant features with high intensity in particular regions of GS 9 and GS 7 tumours, an indication that PCa cells have upregulated mitochondrial uptake of long chain FA to support ATP-generation by ß-oxidation [60]. Moreover, overexpression of carnitine transporter (CPT2) has been reported in primary PCa to support mitochondrial oxidative phosphorylation [61].

DESI imaging has also been applied to compare the relationship between multifocal prostate cancer lipid fingerprints and pathological grade. A recent DESI-MSI study assessed metabolite markers in prostate needle core biopsies in 35 samples from 18 patients by defining pathological regions of interest (ROIs) [62]. Metabolite ions (*n* = 289) were selected from benign and PCa ROIs, and initially a univariate statistical analysis identified metabolites enriched in cancer compared to benign ROIs. This identified significant changes in FA, PE, PI, and PC abundance between the two conditions. Second, metabolites that were differentially abundant between Gleason grade groups were evaluated. Here, they compared grade group (GG)2 and GG3 ROIs and showed that lyso-PEs (16:0 and 18:0) were more abundant in benign tissue whereas dephosphorylated monounsaturated PLs (P-38:1 and P-40:1) and reduced polyunsaturated PLs (O-38:2 and O-40:2), were more prominent with increasing tumour grade. Finally, a logistic regression-based classification model using training/validation samples was built to identify highly sensitive and specific lipid features, which achieved overall balanced accuracies of 97% and 85% in the training and validation sets, respectively.

Another DESI-MSI study investigated the metabolite and lipid composition of 54 fresh–frozen prostate tissue specimens [63]. This work particularly focused on metabolite ions between *m*/*z* 50–200 range found in the Krebs cycle. A least absolute shrinkage and selection operator (Lasso) was used to identify classifiers in 36 tissue samples (18 normal vs. 18 PCa) in a training set and the top 54 peaks were further evaluated in a validation set of 18 samples (10 normal vs. 8 PCa). Interestingly, the Lasso tool showed inferior performance for ions on the lipid *m*/*z* 50–1000 range but better accuracy with inclusion of the Krebs cycle metabolite ions—89% vs. 94% overall agreement. Glucose/citrate ratio was found to be a biomarker that spatially distinguished benign prostatic hyperplasia (BPH) from PCa when ion maps were compared to corresponding histopathological scans [63]. Normal prostatic fluid is rich in citrate [64] and normal prostate cells ostensibly derive citrate from glucose metabolism [65]. The oxidation rate of citrate for ATP production increases as PCa transitions from a Warburg-state glycolysis to FA oxidation [66]. Such approaches are moving towards the clinic; for example, Cook’s group have used DESI-MSI and touch spray mass spectrometry (TS-MS) ionisation to determine surgical margins in men undergoing radical prostatectomy (*n* = 18). DESI-MSI and TS-MSI data had prediction of 97.5% and 96% in discriminating cancer from normal tissue. TS-MS was further validated with accuracy of 92.5% of tumour from normal tissue relative to histopathology and they proposed the technique to be useful for rapid detection of surgical margins [67].

A recent report by Andersen et al. sought to define the metabolomics composition of the different tissue compartments of heterogeneous PCa [68]. This study had a cohort of 15 patients where 45 consecutive tissue sections were analysed in dual MALDI polarities using either 2,5-dihydroxybenzoic acid (DHB) and N-(1-naphthyl) ethylenediamine dihydrochloride (NEDC) matrices. Multivariate pairwise comparisons of cancer, non-cancer epithelium and stroma cell types revealed metabolic alterations in carnitine shuttle indicative of known enhanced fatty acid oxidation and de novo lipid synthesis in PCa cells. Their data also showed reduced levels of metabolites of healthy prostate function (citrate, aspartate, zinc, and spermine) in tumour tissues whilst stroma exhibited higher levels of ADP, ATP, and glucose. A key finding was reduced abundance of LPC (16:0) in cancer compared to non-cancer epithelium consistent with the findings by Goto et al., which further underpins its potential as a prognostic lipid biomarker.

Butler et al. used quantitative lipidomics analyses to characterise the PL profile of prostate tumours compared to benign tissues in a matched (*n* = 21) and independent cohort (*n* = 47) of patient samples [69]. In the matched cohort, ESI-MS/MS analysis of bulk tumour samples revealed multiple PL species across PC, PE, PI, and PS subclasses as significantly correlated with malignancy and the relative proportions of saturated, monounsaturated and polyunsaturated fatty acids content in PLs were also significantly different between normal and tumour patients, most notably featuring increased proportions of monounsaturated PLs. MALDI-MSI revealed distinct variations of lipid features of benign versus malignant epithelial regions using multivariate analysis of pathology annotated clinical samples and was used to validate PE (42:6) and PI (36:4) as positively and negatively correlated with malignancy status, respectively. Additionally, using a patient-derived tissue culture model to probe lipid changes in tumour with response to a current clinical agent enzalutamide, this study further linked phospholipid abundances to ki67 proliferation status where spatial MSI showed that PC (34:1) was decreased after an ex vivo 48-h androgen inhibition challenge. Notably, this work showed, for the first-time using clinical material, how combination of quantitative and spatial lipidomics approaches uncovered aberrant properties of PCa fatty acid synthesis, desaturation, and elongation as new therapeutically rational avenues to control and manage the disease [69].

A novel modification of MALDI-MSI has been the use of ozone to determine the position of fatty acid unsaturation, allowing for the first time more precise identifications of a broader range of lipid species and identifying the canonical and non-canonical enzymatic pathways involved in their production. Young et al. used ozone-induced dissociation (OzID) to explore the diversity of fatty acids in a small number of prostate tissues, and revealed a series of previously unreported species incorporated into PLs in distinct tissue compartments [70]. Importantly, mapping these novel fatty acids across heterogeneous tissues implies that there is plasticity in the various lipid metabolic enzymatic activities throughout multifocal prostate tumours and TME.

## 6. Complementary Spatial Transcriptomics to Map Alterations in Lipid Metabolism in PCa

Many tumour-related changes in lipid profiles reflect alterations in gene regulatory cascades that in turn are driven by oncogenes and tumour suppressors, epigenetics, and other adaptive mechanisms. Hence, complementary transcriptomic analysis has the potential to substantially enrich lipidomic profiles with gene expression data, aiding in the biological interpretation of lipid metabolic changes and in the delineation of pathogenic pathways or potential targets for therapy. Since the inception of DNA microarray technologies, this omics field has evolved dramatically and currently is dominated by next-generation RNA sequencing. Its classical application on tissue extracts shares similar limitations as bulk lipidomics and thus provides a mean gene expression profile across cell populations, even when selected for high tumour content. Single-cell sequencing (scRNA-seq) is a powerful approach to define gene expression heterogeneity at the level of individual cells and is often used as a tool to reveal all cellular subpopulations present in a given tissue [71,72,73]. However, this method involves the dissociation of cells from their original tissue context prior to sequencing, leading to the loss of spatial information.

Traditional technologies in molecular biology, such as situ hybridization (ISH) and immunohistochemistry (IHC), were the first to retain spatial information within tissues by mapping DNA, RNA, and proteins. However, targeted approaches require preselected markers limiting the spatial analysis of a subset of genes and proteins at a time [71,73]. Spatial transcriptomics, on the other hand, enables high-resolution assessment of spatial gene expression across tissue sections, overcoming the limitations associated with tissue homogenisation. This spatially resolved technology has the ability to query the entire transcriptome within a single tissue section in an untargeted way [74].

ST is an overarching term for all methods that assign transcriptomics data to the original location within a tissue region. Based on the size of the tissue that can be examined and the number of genes that can be probed, ST technologies can be subdivided in two main categories: (1) next-generation sequencing (NGS)-based technologies and (2) fluorescence imaging-based approaches comprising in situ sequencing (ISS)-based methods and in situ hybridization (ISH)-based methods. The latter two are targeted techniques and require a priori knowledge of the genes of interest. ISS-based methods directly read out the sequence of transcripts within the tissue [75]. It is based on padlock probing [76] for the revers transcription, followed by rolling-circle amplification [77] and RNA sequencing [75,76,78]. In general ISS-based methods can reach single-cell resolution, are rather low in sensitivity and enable high gene throughput [79]. The second group of imaging-based ST methods are based on ISH technologies, in which a fluorescent imaging probe hybridises sequentially to a target sequence in the tissue [80,81,82,83]. Although ISH-based technologies were rather limited in throughput, the invention of multiplexed error-robust fluorescence ISH (MERFISH) [84,85] and sequential fluorescence ISH (seqFISH) [86] enabled substantial multiplexing localising hundreds of genes in intact tissue. In addition, ISH-based methods allow for subcellular resolution and are highly sensitive [87,88]. In this review, we will mainly focus on NGS-based approaches, which are unbiased spatially resolved methods that query the whole transcriptome from tissue sections. These technologies are well suited for molecular profiling and exploring a new system. Its integration with other unbiased spatial omics technologies, such as spatial lipidomics, will provide a powerful set of tools to characterise prostate cancer heterogeneity and disease processes in intact tissue sections [89].

In 2016, Salmén et al. developed a protocol which combined histological haematoxylin and eosin (H&E) staining with spatially resolved RNA-sequencing which is applicable to fresh-frozen mammalian tissue [71]. Key in this protocol is a micro-array slide, each comprised of 1000 unique barcoded spots (100 μm spot diameter with 200 µm centre-to-centre distance) enabling unbiased investigation of a large tissue area without selecting a specific region or, importantly, a set of genes of interest [90]. Recently, 10X Genomics released the Visium platform, which is an improved version of the technology with increased sensitivity (more than 10,000 transcripts per spot) and resolution (55 μm spot diameter with 100 μm centre-to-centre distance). In the first step, a thin (10 µm) tissue section from fresh-frozen tissue is cut with a cryostat and placed on top of the microarray slide. Then, the tissue is H&E stained and imaged in order for the pathologist to make annotations based on tissue morphology. Following a permeabilisation step, the released mRNA transcripts are spatially captured by hybridisation to the oligo-deoxythymidine (oligo-dT) region of the surface probes and subsequently transcribed to cDNA. After cDNA fragment collection, library preparation and sequencing, an unbiased map of expressed transcripts across the tissue slide is achieved. The spatial transcriptome can then be visualised relative to the histological images [71,73,91].

Currently, there are several commercial systems on the market. For most of these systems, the current lateral resolution of spatial transcriptomics is limited to 50–100 μm, meaning that this method does not produce data on a single-cell level and that the observed expression profile at a given position originates from a potentially heterogenous mixture of adjacent cells, including distinct cell types. The spatial transcriptomic data will provide the location of mRNA transcripts but not the cell types that produced them, while scRNA-seq data characterises each cell type’s expression profile but loses information regarding their position. Therefore, scRNA-seq data are used to deconvolute the spots down to the single cell level, which results in the identification of the cell type population generating the gene expression values within a specific spatial location [71,92]. Thus, a combination of both scRNA-seq and ST data modalities from the same sample can achieve a higher spatial resolution enabling more in-depth tissue analysis.

Applied to the field of PCa, Berglund et al. were the first to measure spatial gene expression profiles using ST technology [11]. Briefly, the pathologist annotated the prostate tissue samples based on cell morphology in its different components, such as stroma, normal and prostatic intraepithelial neoplasia (PIN) glands, immune cells, and cancer. In the ST procedure, read counts of every gene per spot were measured and used to generate a set of factors (cell types), each with a unique expression profile. To identify interactions between the different factors, hierarchical clustering was performed resulting in three main groups. Surprisingly, the inflammatory cells ended up in the same group as PIN and cancer, separated from normal glands. This suggests that inflammation plays an important role in tumour progression. Moreover, an upregulated expression of SPINK1 was found in the cancer region. This finding is consistent with another study revealing that SPINK1 upregulation elicits epithelial-mesenchymal-transition and potentiates cellular-plasticity in patients suffering from androgen receptor (AR)-independent PCa following androgen-deprivation therapy (ADT) [93]. Importantly, some sections annotated as ‘cancer’ by the pathologist belonged to ‘normal or PIN glands’ according to their gene expression profile. Furthermore, it was also observed that some cancer expression regions extended beyond the boundaries of annotated tumour areas. This indicates that a transcriptome-based clinical evaluation could highlight ‘high risk’ areas for the pathologist to pay extra attention to. Finally, this study also provided new insights in pathways with an altered activation in the centre core vs. the periphery of the tumour. Previous studies already revealed that adjacent tissue represents an intermediate state between normal and cancer tissue, but until this study it was never confirmed with spatially resolved data. Berglund et al. found that the tumour centre is dominated by enriched pathways linked to altered cellular metabolism, while the activated pathways in the periphery are mainly related to stress and inflammation [11].

In another study, Ruzzo and Wang assessed the spatially-resolved metabolic networks of the TME in PCa. Metabolic reprogramming is a hallmark of all cancers and is highly influenced by the surrounding environment, such as the presence of blood vessels for nutrients and oxygen. Consequently, heterogenous distributions of blood vessels lead to spatial heterogeneity in the altered metabolism of the TME. It is important to untangle this heterogeneity in order to develop new drug targets for PCa. In this study, ST data were used to identify genes and pathways that where differentially expressed across separate regions of the same primary tumour. Importantly, some of the identified metabolic genes can be targeted with small molecule compounds that are already FDA-approved. Among these was the fatty acid desaturase SCD1, which requires molecular oxygen to perform its function. Hypoxia is a key feature of the TME and therefore these cells need to adapt their metabolism to bypass SCD1, for example by using FADS2 desaturation of fatty acids. However, spatial transcriptomic data in this study revealed that both SCD1 and FADS2 were depleted in the tumour region indicating its dependency on the exogenous uptake of unsaturated fatty acids. Accordingly, a possible way to selectively kill the hypoxic cancer cells would be by pharmacologically inhibiting SCD1 while simultaneously depleting the exogeneous source of unsaturated fatty acids. A second identified target was the prostaglandin transporter SLCO2A1. Prostaglandin is known to have an important function in both angiogenesis and immunomodulation, so inhibiting SLCO2A1 with suramin could be a valuable treatment [14].

## 7. Challenges and New Developments in Spatial Lipidomics and Combined Spatial Omics Approaches

Imaging technologies enabling spatial lipidomics as well as complementary spatial omics approaches are evolving rapidly and are pushing the limits encountered with current platforms. These limits include the sensitivity of detection, unambiguous species annotation, quantification and spatial resolution. Some of these issues are being solved by combining spatial and bulk omics approaches, providing a deeper coverage of lipid profiles.

A critical technical issue in MALDI-MSI is the need to image lowly abundant metabolites and lipids without loss of sensitivity whilst maintaining appreciable spatial resolution. For instance, lipid spectra are typically dominated by signals of high abundant or easily ionisable lipids such as PCs, while other PLs such as PE are hardly detectable. MALDI-2 is a new technique that has been shown to improve ionisation of compounds that pose a challenge in conventional MALDI. In this method, molecules desorbed by the MALDI process are post-ionised by a second pulsed UV laser orthogonally to the direction of the plume created by a first laser. Soltwisch et al. showed that several classes of lipids that are difficult to image with conventional MALDI-MSI appear in the MALDI-2 spectra with signal intensities by up to two orders of magnitude higher [94,95].

Another challenge relates to the small mass range of lipids in complex samples, resulting in the presence of isobaric species, i.e., lipid isotopes with identical or near identical *m*/*z*. This issue can be solved in part using high mass resolution instruments and/or by molecular fragmentation (MS/MS). An interesting complimentary approach is the addition of ion mobility separation, which is a post-ionisation separation technique that distinguishes between ions based on their collisional cross section (CCS) area. This method is one of the few separation techniques that is compatible with surface desorption [96,97]. New additions to these approaches include trapped ion mobility (TIMS) or cyclic ion mobility (CIM). In TIMS [98], ions generated from a single laser shot are accumulated, trapped, and eluted without any loss. In a CIM cell, after multiple passes, the mobility resolution of ions increases allowing ions to be selected for detection. So far, this technology has been applied to separate three distinct isomeric pentasaccharides each with different anomeric configurations (glycosidic linkages) [99]. Both TIMS and CIM devices greatly improve the confidence of metabolomics identification when coupled to high resolution accurate mass MS analysers. This portends unprecedented scales of lipidomics data where ion identity can be ascertained based on retention time, *m*/*z*, MS/MS structural fragments and CCS values. Matched together with the latest processing computer hardware, software and bioinformatics tools, these new MS capabilities are promising to contribute to the discovery of accurate metabolomics markers of PCa and supplement traditional and recent PCa screening and prognosis platforms.

Another challenge relates to the highly complex multidimensional data sets that are generated by spatial omics requiring advanced computational bioinformatics systems and considerable computing power [41]. Many commercial platforms do not have all the necessary statistical tools contained in a single program [100,101], although recently a new program called Lipostar has incorporated spatial metabolomics data analysis [102]. Multi-omics (e.g., transcriptomics, lipidomics, proteomics) should ideally be applied to the same or consecutive tissue slides in order to truly grasp the complexity, heterogeneity, intracellular signalling, and pathophysiological processes underlying prostate cancer progression. For example, in order to validate the findings of spatial transcriptomics, the concordance between gene expression and staining of the corresponding proteins can be assessed in the same tissue regions [11,13]. Furthermore, models that are only based on spatial transcriptomics data are not a direct reflection of metabolic activities, emphasising the need to incorporate other spatially resolved omics techniques, such as lipidomics or proteomics [14]. The combination of spatial omics techniques is also challenged by the different spatial resolutions of the various techniques and need for integration with bulk omics data such as bulk lipidomics and single nuclei RNA-sequencing. Further developments in technological and computational analyses will revolutionise research by providing high-resolution mapping of expression profiles and biomolecules. As a result, it will allow researchers to study in detail how tumour cells interact with adjacent cells or its surrounding ECM. A current stumbling block hindering the progress of spatial omics platforms into clinical settings is that these methods remain relatively low-throughput, expensive, technically challenging, and still may benefit from higher spatial resolution [13]. An enduring challenge that is common to all multi-omics approaches is that advanced methods are needed to effectively integrate data across different modalities to improve both diagnostics and therapy.

## 8. Conclusions

Emerging knowledge on the rewiring of lipid metabolism in PCa, along with recent developments in spatial omics approaches to map lipid profiles at a near single cell level in intact tissue, provide hitherto unseen insights into the spatial heterogeneity of PCa that likely underlies differential disease progression and therapy response. The resulting molecular mapping of the tumour ecosystem generates unique insight into altered cellular functions and interactions in situ with the potential to identify novel disease-specific targets and biomarkers. The rapid adoption of spatial omics approaches in the clinical pathological setting and the cost-effectiveness of lipid-based MS imaging compared to other spatial omics approaches has the potential to transform and refine routine clinical decision-making, resulting in better and more personalised patient outcomes.

## Figures and Tables

**Table 1:** Key studies using MSI as a spatial tool for prostate cancer metabolomics and lipidomics.

Author	Method	Findings
Butler et al., 2021	ESI-MS/MS MALDI-MSI	Association of lipid profiles to malignancy status in clinical biopsies and lipid changes in response to metabolic targeting agents
Young et al., 2021	MALDI-MSI OzID	Isomer-resolved lipidomics detects non-canonical fatty acids (reflecting different desaturase activities) present in different regions of the PCa TME, providing support for discrete localisation of desaturase enzymes
Andersen et al., 2021	MALDI TOF MSI	Lipid and metabolite composition was distinct between stromal, non-cancerous epithelium, and PCa. Lysophospholipids had lower abundance in PCa versus non-cancerous epithelium, while PE and PI lipids were higher in PCa.
Randall et al., 2019	MALDI FT-ICR MSI, MALDI TOF MSI	Prostate tumours can be differentiated using different Gleason grades based on metabolomic differences
Morse et al., 2019	DESI-MSI	Logistic regression and PCA/LDA model of lipid and metabolite classifiers can reliably identify cancer and distinguish Gleason grade groups
Banerjee et al., 2017	DESI-MSI	LASSO model identified glucose and citrate as predictors of PCa and normal tissue
Wang et al., 2017	MALDI FT-ICR	Increased energy charge and low abundance of neutral triglycerides in cancerous tissue
Goto et al., 2015	MALDI-MSI	LPC (16:0) and SM (d18:1/16:0) were lower in tumour compared to benign epithelium. LPA (16:0) was an independent predictor of biochemical recurrence after radical prostatectomy
Goto et al., 2014	MALDI-MSI	PI species were more abundant in cancer compared to benign epithelium: PI (18:0/18:1) PI (18:0/20:3) PI (18:0/20:2)
